# Value of animal sepsis research in navigating the translational labyrinth

**DOI:** 10.3389/fimmu.2025.1593342

**Published:** 2025-04-15

**Authors:** Haichao Wang, Alfred Ayala, Monowar Aziz, Timothy R. Billiar, Clifford S. Deutschman, Samithamby Jeyaseelan, Daolin Tang, Ping Wang

**Affiliations:** ^1^ The Feinstein Institutes for Medical Research, Northwell Health, Manhasset, NY, United States; ^2^ Department of Molecular Medicine, Donald and Barbara Zucker School of Medicine at Hofstra/Northwell, Hempstead, NY, United States; ^3^ Division of Surgical Research, Brown University Health-Rhode Island Hospital, Providence, RI, United States; ^4^ Department of Surgery, the Warren Alpert School of Medicine at Brown University, Providence, RI, United States; ^5^ Department of Surgery, University of Pittsburgh, Pittsburgh, PA, United States; ^6^ Department of Pathobiological Science, School of Veterinary Medicine, Louisiana State University, Baton Rouge, LA, United States; ^7^ Department of Surgery, UT Southwestern Medical Center, Dallas, TX, United States

**Keywords:** sepsis, animal model, human therapy, therapeutic targets, rheumatoid arthritis

## Introduction

Sepsis is a life-threatening organ dysfunction caused by a dysregulated host response to infection (1), as manifested by early activation of both pro- and anti-inflammatory responses (2), along with major alterations in non-immunologic pathways such as cardiovascular, neuronal, autonomic, hormonal, bioenergetic, metabolic, and coagulation (3). It accounts for almost 20% of total deaths worldwide (4), and annually costs more than $60 billion in the U.S. alone. The onset of the disease and the intricate interplay of various immune cells, inflammatory mediators, signaling pathways, and organ systems makes studying sepsis in humans ethically and logistically challenging. This necessitates the use of animal models to systematically dissect its intricate pathophysiology and evaluate potential therapies in a controlled setting, which has contributed to developing and implementing clinical therapies for other inflammatory diseases, notably rheumatoid arthritis.

## The indispensable role of animal models in sepsis research

Animal models allow researchers to manipulate key variables such as infection type and severity, intervention timing, and the genetic background (e.g., gene knockout or knock in strategy) of experimental animals (5–8). This level of control enables researchers to isolate the effects of specific interventions and identify potential therapeutic targets, such as tumor necrosis factor (TNF) (9), high mobility group box 1 (HMGB1) (10), cold-inducible RNA-binding protein (CIRP) (11), sequestosome-1 (SQSTM1) (12), and procathepsin L (pCTS-L) (13). Moreover, these models allow for tracking the temporal progression of sepsis from initial insult to subsequent organ dysfunctions (14) and eventual outcomes (7, 8), offering invaluable insight into the complex interplay of multiple pathophysiological processes (15, 16), including hyperinflammation (17), immunocoagulation (18, 19), pyroptosis-mediated immune cell death (20), and immunosuppression (21, 22). Given the influence of comorbidities and other factors on disease progression and treatment response in human sepsis, it is essential to incorporate comorbidities (e.g., diabetes, hypertension, or coronary artery disease) and pre-existing injuries (e.g., smoke inhalation occurring in burn patients) into animal modeling, thereby improving the translatability of experimental findings into future clinical therapies (6–8, 23).

## The challenges and complexities of translation

Despite advancements in understanding sepsis pathophysiology, translating preclinical findings into effective human therapies remains challenging, as exemplified by the failure of anti-TNF antibodies in clinical trials (3, 24). However, attributing this translational gap solely to the limitations of animal models is an oversimplification (6), because the inherent complexity and heterogeneity of human sepsis, coupled with challenges in clinical trial design, also contribute to this difficulty.

Animal models typically use a single, standardized insult in genetically homogeneous animals. However, this genetic and environmental homogeneity of laboratory animals contrasts sharply with the genetic and environmental diversity of human populations, as well as the variety of infections in clinical sepsis (8, 25). The inherent heterogeneity in septic patients is further compounded by other factors such as age, sex, underlying health conditions (comorbidities), environmental exposure/history, and time to treatment initiation (2). Because patient variability often creates a broad spectrum of pathophysiological endotypes, it is important to develop animal models to recapitulate some human sepsis endotypes. While comprehensive immune profiling (cytokine/chemokine levels, immune cell function, gene expression) can potentially characterize “endotypes” in animal models, their accuracy in reflecting human sepsis endotypes (such as hyper- or hypo-inflammatory states) remains unclear (6–8), presenting challenges for translational research. Thus, the failure of identifying and recruiting homogenous patient subgroups in previous clinical trials might have diluted treatment effects due to potential outcome variations (8, 23, 26).

In addition, potential differences in immune responses between animals and humans may pose another significant challenge (27). Although genomic comparisons between mouse models and human sepsis have revealed significant similarities (28, 29), they also highlight some noticeable differences (30), underscoring the complexity and difficulty of extrapolating experimental findings across species (**Table 1**). Therefore, developing more diverse and sophisticated animal models that incorporate polymicrobial infections, comorbidities, and genetic variability is crucial to improving translatability (7, 8). For example, a refined murine sepsis model that adhered the Minimum Quality Threshold in Pre-Clinical Sepsis Studies (MQTiPSS) guidelines (5, 31) by incorporating daily chronic stress closely recapitulated the genomic and phenotypic responses observed in human surgical sepsis (32). Similarly, humanized mice that express human genes or possess a humanized immune system may similarly offer a more promising approach to enhance translatability (7, 8, 33). Conversely, stratifying patients based on specific biomarkers (34, 35) indicative of the unique pathobiology one is hoping to modify, along with relevant clinical parameters beyond overall mortality, should similarly enhance the precision and power of future clinical trials (3, 35–37).

**Table 1:** Strengths and weaknesses of various animal models of sepsis.

Animals	Strengths	Weakness
Zebrafish	Cost-effective Offers excellent visualization platform	Overly simplified immune system limits translatability
Rodents (Mice and Rats)	Affordable Genetically manipulable	Small size and differing immune responses hinder clinical relevance
Guinea Pigs	Physiological similarities to humans, particularly in cardiovascular and coagulation systems	Lack extensive genetic tools
Pigs and Sheep	Greater physiological and immunological resemblance to humans Enable the use of advanced organ support technologies for real-time monitoring of physiological and immunological dysregulations	Higher cost Longer experimental duration Species-specific differences Restricted genetic manipulation Heightened ethical concerns
Non-human Primates	Closest physiological and immunological match with human	Higher costs Significantly restricted by ethical concerns
Human *ex vivo* Models	Replicate the complex interplay between immune cells, endothelial cells, and pathogens within a controlled microenvironment Investigate patient-specific responses to identify individualized responses and to tailor personalized therapies	Cannot fully replicate the intricate interplay of factors within a living organism Experimental duration is constrained by tissue/cell viability *ex vivo* Face challenges such as ethical approvals, logistical coordination, and cost/expertise limitations

The rationale for targeting TNF in sepsis stemmed from its early and prominent role in initiating the inflammatory response (9, 38). However, sepsis is a highly heterogeneous syndrome characterized by variable timing for the release and pathogenic actions of various cytokines (17). While TNF may be critical in the initial hyperinflammatory phase, its importance diminishes over time. Therefore, blocking it at a wrong time could even be detrimental to the host by impairing essential immunities needed for pathogen clearance (39–42). Accordingly, shifting the focus to some later-acting mediators such as HMGB1 (10) and pCTS-L (13), which have relatively wider therapeutic windows (43), may present a more promising avenue for future sepsis trials. Given the multifaceted nature of sepsis, combinatorial therapies targeting multiple mediators may be more effective than targeting a single cytokine. Such combination therapies, if tailored to the specific cytokine profile and disease stage of individual patients, may offer a more personalized and effective approach to sepsis treatment (8, 35, 44), unlocking the therapeutic potential of cytokine-targeting therapies for this devastating condition.

The dynamic nature of sepsis requires timely interventions and optimal dosing regimens (45, 46), which are also difficult to translate from animal models to human clinical trials. Simplified dosing regimens used in animal models (32) often struggle to capture the complex pharmacokinetics and pharmacodynamics observed in humans (3). Beyond the heterogeneity of sepsis patients, interspecies differences in these kinetic and dynamic parameters further complicate translation, necessitating personalized dosing algorithms based on individual patient characteristics such as age, sex, weight, comorbidities, and disease severity.

## The unexpected value of animal sepsis research in human therapies

Although directly translating animal sepsis research to human sepsis therapies remains challenging, the knowledge gained from animal models has advanced treatments for other inflammatory diseases, such as rheumatoid arthritis (RA) (47), Crohn’s diseases (48), and ulcerative colitis (49). RA, a chronic autoimmune disease affecting 0.5–1% of the global population (50), shares unexpected commonalities with sepsis in its inflammatory pathways, particularly the involvement of TNF (51, 52) and other cytokines. The identification of TNF as an early mediator of sepsis, largely through animal models (9, 38), paved the way for the development of anti-TNF biologics like infliximab, etanercept, and adalimumab (53, 54) as cornerstone therapies for RA (53, 55), Crohn’s diseases (48), and ulcerative colitis (49). These success stories have exemplified the broader impact of sepsis research using animal models, extending beyond sepsis itself to benefit patients with other inflammatory conditions. It highlights the value of fundamental research in revealing unexpected connections between seemingly disparate fields and potentially driving significant therapeutic advances for many inflammation disorders.

## The path forward: refining models and experimental approaches

Animal models remain indispensable in sepsis research, providing a controlled setting to unravel complex pathophysiological mechanisms, identify therapeutic targets, and evaluate novel interventions. While acknowledging their limitations and actively refining these models is crucial, abandoning animal research would be a short-sighted setback hindering scientific progress (6). Therefore, the future of sepsis research still hinges on developing more sophisticated and clinically relevant animal models that incorporate age, sex, polymicrobial infections, comorbidities (e.g., diabetes, hypertension, or coronary artery disease), pre-existing injuries (e.g., smoke inhalation in burn patients) (56), and genetic diversity, thereby better reflecting the complexity and heterogeneity of human sepsis. Animal models of sepsis can be developed in a vast array of different animal species (**Table 1**), but each species possesses unique strengths and weaknesses that influence the translatability of research findings. Therefore, model selection depends on the specific research question and a balanced assessment of species-specific strengths and weaknesses. For instance, a multi-species approach, strategically incorporating larger animal models like pigs or non-human primates when necessary, might be needed to bridge this species gap.

Traditional animal sepsis studies rely on limited number of physiological and biochemical markers, and thus lack the granularity to capture the complex molecular landscape of clinical sepsis. It is thus critical to refine animal sepsis models (57) by integrating transcriptomic, epigenomic, metabolomic, and proteomic analyses across various body compartments at single-cell level (58). Comparing these multi-omics profiles between animal models and human sepsis patients will allow for rigorous model validation, ensuring an accurate reflection of dysregulated molecular pathways of human sepsis (32). This improved approach may also help identify key pathways and biomarkers conserved across species, guiding relevant therapeutic target selection to improve the predictive power of preclinical studies.

To fully replicate organ dysfunctions characteristic of human sepsis, it is paramount to integrate organ support technologies (e.g., mechanical ventilation, fluid resuscitation, and vasopressor support) into larger animal models by creating “ICU-like” experimental conditions that allow real-time monitoring and modulation of immunological and physiological parameters that mirror clinical sepsis management. Large animal models, like pigs and sheep (56, 59), offer a unique platform for sepsis research due to their larger size, as well as immunological and physiological similarities to humans (e.g., heart rate, blood pressure, and lung mechanics), enabling the use of advanced organ support technologies for real-time monitoring of immune and physiological dysregulations during sepsis (**Table 1**). However, these models are also limited by higher cost, longer experimental duration, species-specific differences, restricted genetic manipulation, and heightened ethical concerns (**Table 1**).

To overcome the translational limitations of animal models in sepsis research, several human *ex vivo* models have also been developed, including **i**) whole blood assays (60); **ii**) precision-cut tissue slices (lung and others) (61); **iii**) human blood-perfused organ models (62, 63); and **iv**) miniaturized “organ-on-a-chip” systems (64, 65). These models can replicate the complex interplay between immune cells, endothelial cells, and pathogens within a controlled microenvironment, offering a more human-relevant platform for studying sepsis (**Table 1**). Another key strength is their capacity to dissect patient-specific responses, using samples from diverse cohorts (varying ages, comorbidities, genetic backgrounds) to identify individualized responses and to potentially tailor personalized therapies. Despite these advantages, human *ex vivo* models suffer from many limitations (**Table 1**), such as the inability to fully replicate *in vivo* complexity, restricted experimental duration due to *ex vivo* tissue/cell viability, and challenges in obtaining/utilizing human samples, including ethical approvals, logistical coordination, and cost/expertise limitations.

## Conclusions

Refining animal models of sepsis requires a multifaceted approach encompassing: **1**) the development of more sophisticated and clinically relevant animal models that incorporate age, sex, polymicrobial infections, and comorbidities; **2**) the integration of organ support technologies and multi-omics calibration; **3**) the strategic use of multiple species; and **4**) the selection of multiple more feasible therapeutic targets including HMGB1, CIRP, and pCTS-L. By implementing these refinements, future animal research can more accurately reflect the complexity of human sepsis, thereby enhancing the predictive validity of preclinical studies and accelerating the development of effective therapies for this devastating condition. In addition to refining animal models, improving clinical trial design through better patient stratification based on biomarkers and cytokine profiles may be equally important. The continued pursuit of knowledge through well-designed animal models, coupled with rigorous clinical research, holds the key to unlocking effective therapies for sepsis and other inflammatory diseases. The unexpected success of anti-TNF therapies in RA, born from sepsis research, serves as a powerful testament to the value of animal research.
